# Identification of a human estrogen receptor α tetrapeptidic fragment with dual antiproliferative and anti-nociceptive action

**DOI:** 10.1038/s41598-023-28062-9

**Published:** 2023-01-24

**Authors:** Baptiste Jouffre, Alexandre Acramel, Mathilde Belnou, Maria Francesca Santolla, Marianna Talia, Rosamaria Lappano, Fariba Nemati, Didier Decaudin, Lucie Khemtemourian, Wang-Qing Liu, Marcello Maggiolini, Alain Eschalier, Christophe Mallet, Yves Jacquot

**Affiliations:** 1 NEURO-DOL Basics and Clinical Pharmacology of Pain, INSERM - UMR 1107, University of Clermont Auvergne, 63000 Clermont-Ferrand, France; 2 Faculty of Medicine, ANALGESIA Institute, 63000 Clermont-Ferrand, France; 3 CiTCoM, CNRS - UMR 8038, INSERM 1268, Faculty of Pharmacy of Paris, University of Paris Cité, 75270 Paris Cedex 06, France; 4 Department of Pharmacy, Institut Curie, 75248 Paris, Cedex 05, France; 5 Laboratoire des Biomolécules (LBM), CNRS - UMR 7203, Sorbonne Université, Ecole Normale Supérieure, PSL Research University, 75252 Paris Cedex 05, France; 6 Department of Pharmacy, Health and Nutritional Sciences, University of Calabria, Rende, Italy; 7 Preclinical Investigation Laboratory (LIP), Department of Translational Research, Institut Curie, PSL Research University, 75248 Paris Cedex 05, France; 8 Department of Medical Oncology, Institut Curie, 75248 Paris Cedex 05, France; 9 Institute of Chemistry and Biology of Membranes and Nanoobjects (CBMN), CNRS - UMR 5248, Institut Polytechnique Bordeaux, University of Bordeaux, 33600 Pessac, France

**Keywords:** Biophysics, Endocrinology

## Abstract

The synthetic peptide ERα17p (sequence: PLMIKRSKKNSLALSLT), which corresponds to the 295–311 region of the human estrogen receptor α (ERα), induces apoptosis in breast cancer cells. In mice and at low doses, it promotes not only the decrease of the size of xenografted triple-negative human breast tumors, but also anti-inflammatory and anti-nociceptive effects. Recently, we have shown that these effects were due to its interaction with the seven-transmembrane G protein-coupled estrogen receptor GPER. Following modeling studies, the C-terminus of this peptide (sequence: NSLALSLT) remains compacted at the entrance of the GPER ligand-binding pocket, whereas its N-terminus (sequence: PLMI) engulfs in the depth of the same pocket. Thus, we have hypothesized that the PLMI motif could support the pharmacological actions of ERα17p. Here, we show that the PLMI peptide is, indeed, responsible for the GPER-dependent antiproliferative and anti-nociceptive effects of ERα17p. By using different biophysical approaches, we demonstrate that the NSLALSLT part of ERα17p is responsible for aggregation. Overall, the tetrapeptide PLMI, which supports the action of the parent peptide ERα17p, should be considered as a hit for the synthesis of new GPER modulators with dual antiproliferative and anti-nociceptive actions. This study highlights also the interest to modulate GPER for the control of pain.

## Introduction

The P^295^LMIKRSK^302^ and K^303^NSLALSLT^311^ fragments of the human estrogen receptor α (ERα) belong to its autonomous activation function AF2a (hinge region, D domain) and to its ligand-binding domain (E domain), respectively^1^. The K^299^RSKK^303^ basic motif corresponds to the third ERα nuclear localization signal (NLS)^1,2^. Following crystal structures, the 295–311 region of ERα is partially folded into polyproline II (PPII)^3^, an extended conformation usually found in protein–protein interaction modules^4,5^. Accordingly, it participates in the recruitment of Ca^2+^-calmodulin^6,7^, Hsp70^8^ and the ubiquitin-protein isopeptide ligase E6-associated protein (E6AP), an E3 ligase catalyzing the ubiquitination of ERα^9^. It associates with ERα, itself, suggesting that it could participate in homodimerization process^10^. The residues Lys-299, Lys-302, Lys-303, Ser-305 and Thr-311 are targeted by post-translational modifications^11^ such as SET7-mediated methylation^12,13^, acetylation^14^, phosphorylation^14,15^, ubiquitination^16^ and SUMOylation^17^. Interestingly, the ERα mutant lacking the residues 295 to 311 (ERαΔ 295–311) displays constitutive transcriptional activity^7^. Likewise, the pre-malignant mutant K303R is closely linked to the phosphorylation of the Ser-305 and, therefore, to poor clinical outcome by conferring to mammary cancer cells estradiol hypersensitivity and resistance to tamoxifen^18–20^. Thus, a key role of the ERα 295–311 region in the control of transcription seems highly likely. In the light of these observations, we have embarked in the study of the pharmacological profile of the peptide corresponding to the 295–311 region of ERα and named ERα17p (sequence: H_2_N-PLMIKRSKKNSLALSLT-COOH).

In physiological conditions (complete serum) and at the concentration of 10 μM, ERα17p triggers membrane-initiated pro-apoptotic events, in both ERα-positive and -negative breast cancer cell lines with, however, a preference for ERα-positive cancer cells^21^. It decreases the ratio Bcl-_xl_/Bax, increases cleaved caspase-9 and induces a redistribution of actin through a mechanism requiring PI3K, ROCK and p38 MAPK, depending on cell line^21,22^. Moreover, ERα17p induces a proteasome-dependent decrease of the levels of the G protein-coupled estrogen receptor (GPER), of activated EGFR and ERK1/2 and, therefore, of the amount of c-fos^23^. Briefly, the estrogen receptor GPER, which belongs to the rhodopsin (class A) family of GPCRs, is distributed in multiple tissues where it exerts both physiological and pathological actions^24–29^. It works in concert with ERα and its alternative spliced isoform ERα36, as well as with the growth factor receptors EGFR and IGF-1R^30–32^. As strongly suggested by staining and docking studies, the pharmacological effects of ERα17p result from its binding within the ligand-binding site of GPER (Kd in the micromolar range)^23,33^. Remarkably, ERα17p induces at low doses and in short times (1.5 mg/kg body weight, three times a week for four weeks) a decrease of about 50% of the size of xenografted triple negative breast tumors, in mice^21^. Likewise and from 2.5 mg/kg, it supports anti-inflammatory and analgesic actions^34^. Hence, the synthetic peptide ERα17p, which corresponds to the 295–311 fragment of ERα, could open new clinical perspectives, particularly in oncology where inflammation and pain are of prime importance^35^.

In previous computational studies, we have shown that the C-terminus of ERα17p (sequence: NSLALSLT) was compacted at the entrance of the pocket, whereas its PLMI counterpart was engulfed in depth within the extracellular GPER ligand-binding site^23^. Such an observation suggests that the PLMI motif could support the pharmacological action of ERα17p and that the NSLSLALT sequence could be responsible for its propensity to form aggregates^36,37^. Here, we confirm that the peptide fragment NSLALSLT is indeed responsible for aggregation and that the PLMI counterpart induces GPER-dependent antiproliferative action. In a murine inflammatory pain model, a maximum analgesic action is reached at low dose of ERα17p. These effects are recovered with the analogue H_2_N-PLMI-COOH, which remains active at higher concentration.

## Results

### The formation of ERα17p amyloid fibrils depends on peptide concentration and pH value

Fluorescence spectroscopy and ^1^H-NMR were used to study at different peptide concentrations the time-course formation of ERα17p aggregates, in acidic (pH 3.4), physiological (pH 7.4) and basic (pH 9.1) conditions. Importantly, the tested pH values were kept at distance of the pI of ERα17p (calculated pI = 11.8) to avoid precipitation.

**Figure 1:**
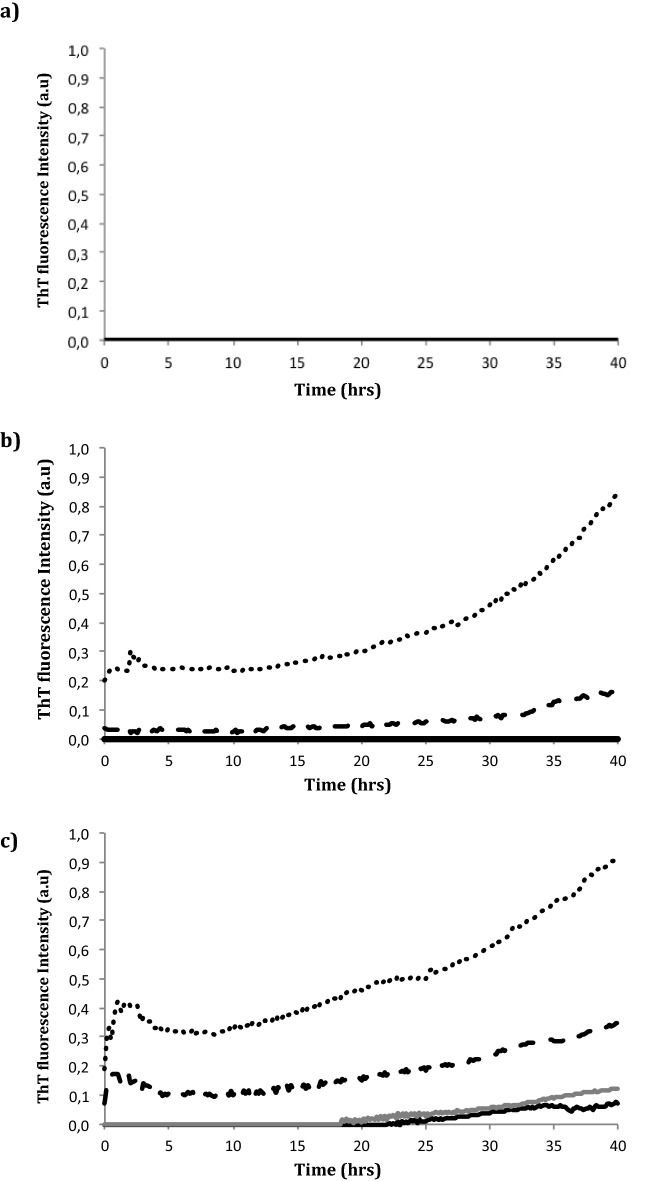
Kinetics of formation of ERα17p amyloid fibrils by ThT fluorescence spectroscopy, at various peptide concentrations (in μM) and pH values. Experiments are tested in water at four peptide concentrations, i.e. at 10 (black curve), 25 (grey curve), 50 (black dashed line) and 100 μM (black dotted lines), in the presence of ThT 10 μM. (**a**) pH 3.4 (0.2 M glycine HCl in water), (**b**) pH 7.4 (0.2 M KH_2_PO_4_/0.2 M K_2_HPO_4_ in water) and (**c**) pH 9.1 (0.2 M glycine NaOH in water). Excitation and emission wavelengths are 440 and 485 nm, respectively. Fluorescence intensity is expressed in arbitrary units (a.u.) and time in hours. Data are the means of experiments performed in triplicate. Each experiment is carried out over 40 h.

First, the formation of ERα17p aggregates was followed over 40 h by thioflavin T (ThT) fluorescence spectroscopy^38,39^. No aggregation was observed in acidic conditions (Fig. 1a). At the concentrations of 50 and 100 μM and at pH 7.4 (Fig. 1b) and 9.1 (Fig. 1c), an exponential time-dependent increase of the formation of aggregates was recorded. At 10 and 25 μM and at pH 9.1, a delay of 18 h was observed (Fig. 1c).

**Figure 2:**
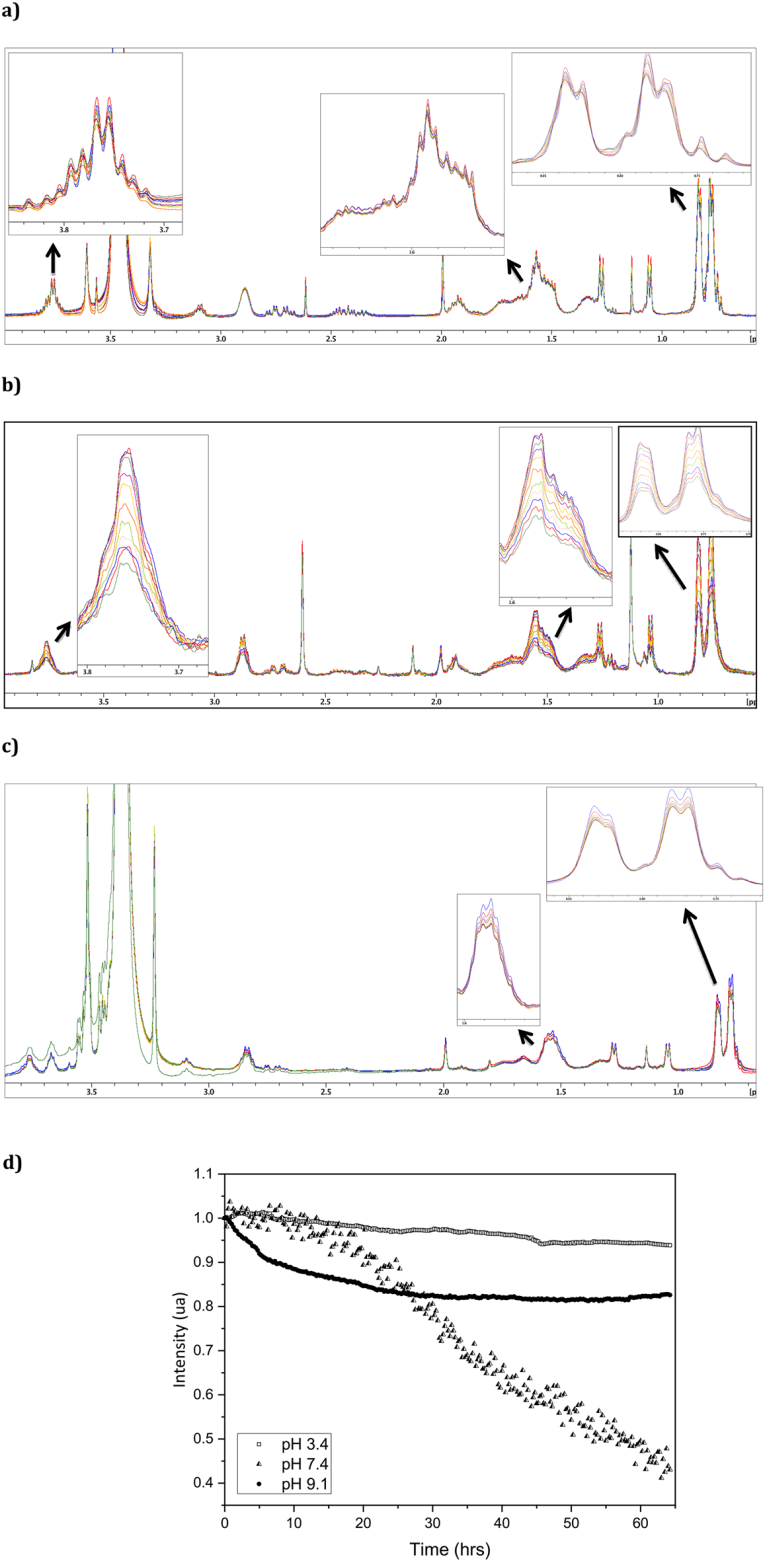
Evolvement of the intensity of the ERα17p aliphatic proton signals as a function of time, by 1D ^1^H-NMR (500 MHz). Aliphatic proton signals of highest intensity (0.80, 0.90, 1.50 and 3.60 ppm) are used to follow aggregation. Each experiment (8 scans/15 min.: 258 points over 65 h) is performed at (**a**) pH 3.4 (0.2 M glycine HCl in water), (**b**) 7.4 (0.2 M KH_2_PO_4_/0.2 M K_2_HPO_4_ in water) and (**c**) 9.1 (0.2 M glycine NaOH in water), at 298 K and with peptide samples of 100 μM in water. (**d**) Evolvement of the intensity of the proton signals as a function of time, at pH 3.4, 7.4 and 9.1.

To confirm previous results, the evolvement of the intensity of ERα17p aliphatic proton signals at 0.80, 0.90, 1.50 and 3.60 ppm was followed over a longer period (65 h) at pH 3.4, 7.4 and 9.1 (peptide concentration: 100 μM), by liquid-state ^1^H-NMR spectroscopy^40,41^. No significant change was found at pH 3.4, even after 65 h (Fig. 2a,d). At pH 7.4, an immediate gentle decrease of the intensity of the proton signals, followed by a steeper negative curve slope (about 40% of the total amount of peptide remained soluble, Fig. 2b,d), was observed. At pH 9.1, a decay of the proton signals followed by a plateau was recorded (Fig. 2c,d).

**Figure 3:**
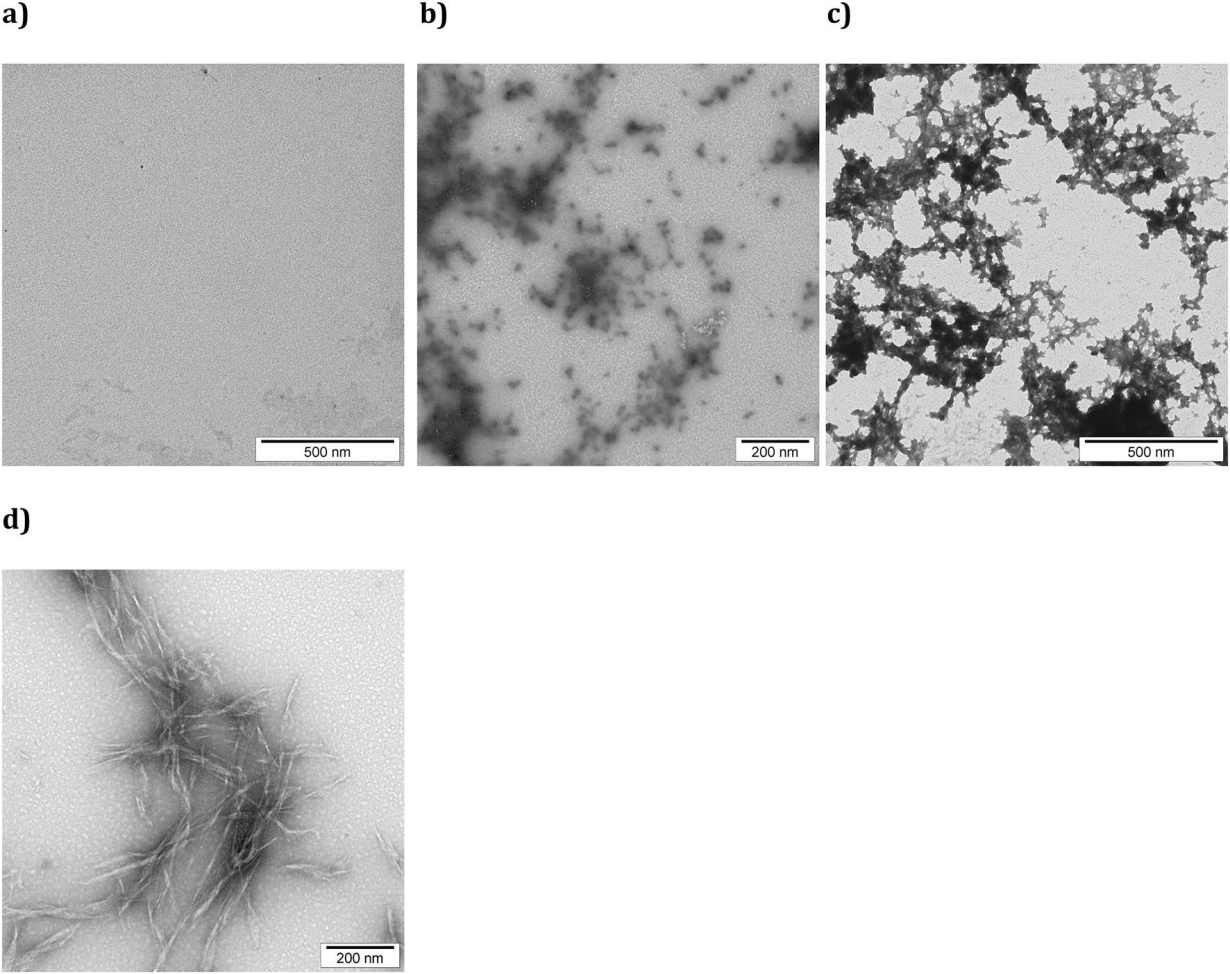
TEM images of ERα17p amyloid fibrils. ERα17p is incubated over 48 h at the concentration of 100 μM at the pH values of (**a**) 3.4 (glycine HCl 0.2 M in water), (**b**) and (**c**) 7.4 (0.2 M KH_2_PO_4_/0.2 M K_2_HPO_4_ in water) and (**d**) 9.1 (0.2 M glycine NaOH in water).

The morphology of the ERα17p aggregates was studied by transmission electron microscopy (TEM) after an incubation period of 48 h (peptide concentration: 100 μM). Again, no aggregate was detected in acidic conditions (Fig. 3a). At pH 7.4, granulations resembling to a dense network of prefibrillar amyloid oligomers (Fig. 3b) and beads with a diameter ranging from 10 to 20 nm on a string and meshworks of packed fibrils assimilated to protofibrils (Fig. 3c) were evidenced^42–44^. As shown in the Fig. 3d, an unambiguous entanglement network of mature amyloid fibrils was observed at pH 9.1 with a length ranging from 100 to 200 nm to > 1 μm, depending on the incubation period (1 and 48 h, respectively).

### The C-terminus of ERα17p supports the formation of fibrils

**Figure 4:**
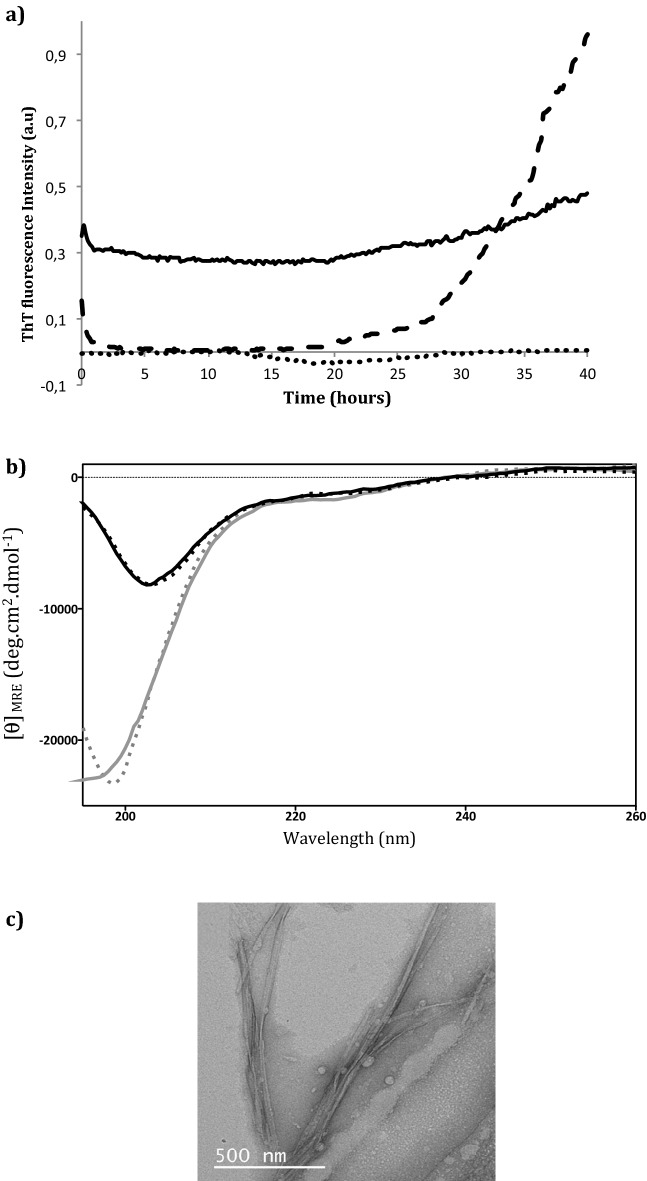
Study of the formation of peptide **1** fibrils. (**a**) Kinetics of formation of fibrils from peptide **1** (black dashed line) and peptide **2** (black dotted line) by ThT fluorescence spectroscopy. Experiments are carried out in water at the concentration of 100 μM and at pH 9.1 (glycine NaOH 0.2 M). ThT is used at the concentration of 10 μM. ERα17p (black line) is the reference. Excitation and emission wavelengths are 440 and 485 nm, respectively. Fluorescence intensity and time are expressed in arbitrary units (a.u.) and hours, respectively. Data are the means of experiments performed in triplicate. Each experiment is carried out over 40 h. (**b**) Kinetics of formation of peptide **1** aggregates recorded by CD. Experiments are performed after an equilibration period of 5 min, at 25 °C and pH 9.1, within the wavelength range 190 and 260 nm and at different incubation times, i.e., at 0 h (grey curve), 5 h (grey dotted lines), 22 h (black curve) and 28 h (black dotted line). CD unit is expressed as mean residue ellipticity [*θ*]_MRE_ (in deg.cm^2^.dmol^−1^). (**c**) TEM image of peptide **1** fibrils at the concentration of 100 μM, after 48 h incubation and at pH 9.1 (glycine NaOH 0.2 M in water).

The propensity of the peptides corresponding to the C- and N-termini of ERα17p, i.e. of the peptides **1** (H_2_N-NSLALSLT-COOH, calculated pI = 5.98) and **2** (H_2_N-PLMI-COOH, calculated pI = 5.98), respectively, to form aggregates was explored. This study was performed over 40 h by using ThT fluorescence spectroscopy. All experiments were carried out with 100 μM of peptide and at pH 9.1. ERα17p was used as the reference. As shown in the Fig. 4a, the peptide **2** failed to show aggregates. In contrast, an exponential increase of the fluorescence signal was recorded with peptide **1**, from 15 to 40 h.

The secondary structure of the soluble pool of the peptide **1** was studied by circular dichroism (CD), at pH 9.1. To this aim, the peptide concentration was fixed at 100 μM. A CD signature relevant to a random coil conformation (typical strong negative maximum at ~ 198 nm, ππ* electronic transition) was recorded throughout a period of 28 h (Fig. 4b). Between 5 and 22 h, the intensity of the negative maximum decreased from − 24,000 to − 7700 deg. cm^2^.dmol^−1^, respectively. A bathochromic effect of 6 nm was concomitantly observed with a decrease of 70% of the soluble pool of **1**. As shown by TEM, this peptide formed stacked fibrils of 1.5 μm length and a thickness of 20 nm (Fig. 4c).

### The peptidic sequence PLMI exerts an enhanced GPER-dependent antiproliferative action when compared to the parent peptide ERα17p

**Figure 5:**
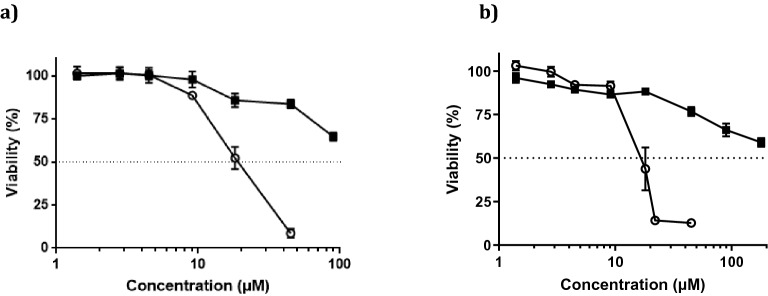
Concentration-dependent effects of ERα17p (-■-) and peptide 2 (-○-) on cells viability (in %), in (**a**) MCF-7 and (**b**) HS578T breast cancer cells. Concentrations are expressed in μM. Each experiment is performed in triplicate (N = 3). Standard errors of the means (± SEM) are specified.

The peptides ERα17p and **2** were evaluated on cell viability. The peptide **1** being insufficiently soluble, it was excluded from biological assays. The two GPER-positive breast cancer cell lines MCF-7 (ER + , PR + , Her2 + , GPER+^45^) and HS578T (ER-, PR-, Her2-, GPER+^45^) were used as model systems (control: ERα17p). In both cell lines and from 10 μM, the peptide **2** was able to provoke a more pronounced decrease of cell survival (IC_50_ ~ 18 μM), when compared to ERα17p (Fig. 5a,b).

**Figure 6:**
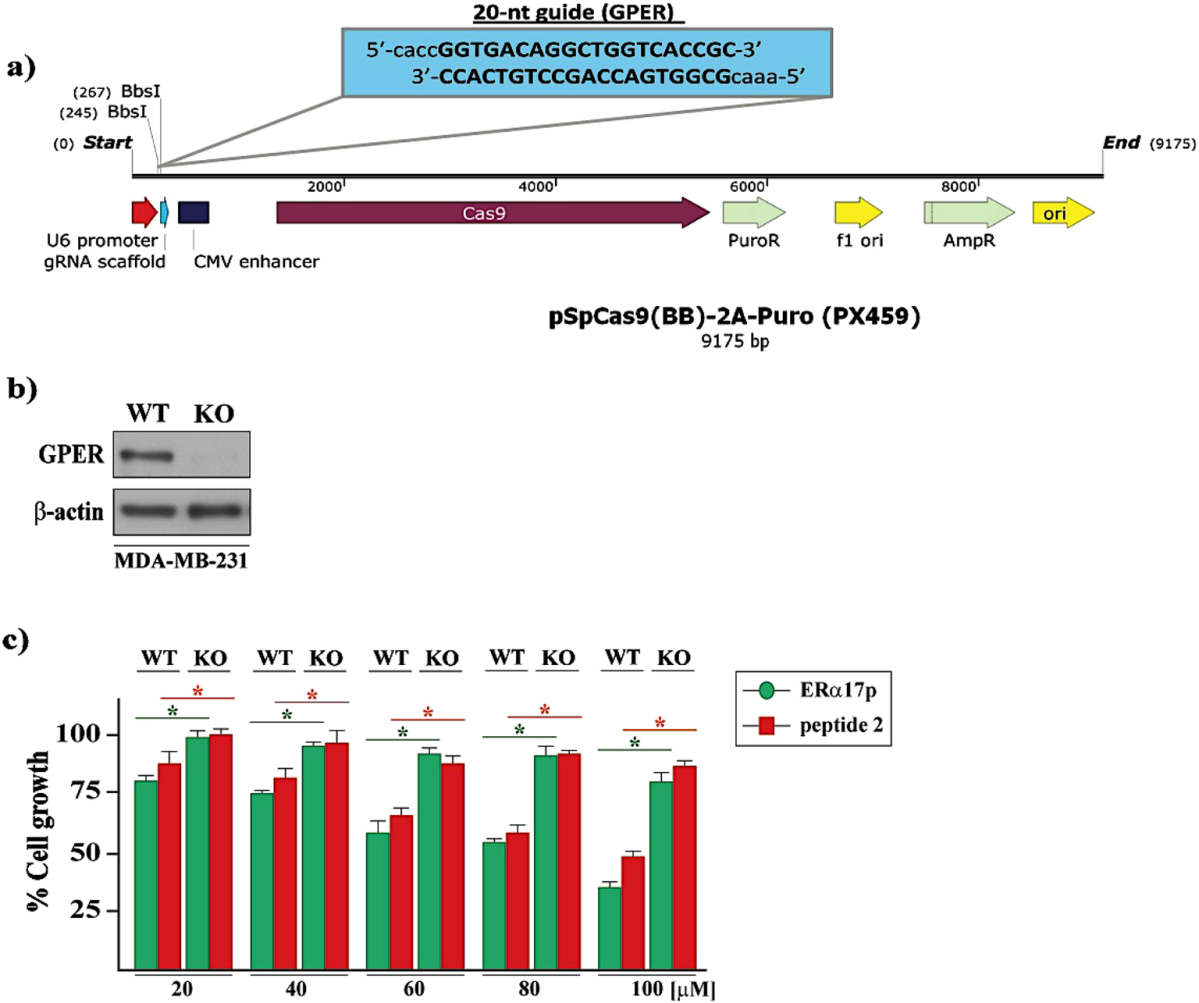
(**a**) Schematic illustration of the sgRNA sequence cloned into the pX459 vector used to generate GPER knockout (KO) in MDA-MB-231 cells. (**b**) Protein expression levels of GPER in GPER (WT) and GPER (KO) MDA-MB-231 cells, as evaluated by western blotting analysis. β-Actin served as loading control. Immunoblots shown are representative of three independent experiments. Original blots are presented in Supplementary Fig. 1. GPER (WT) and GPER (KO) (**c**) MDA-MB-231 cells are treated for 72 h with either vehicle or increasing concentrations of ERα17p and peptide **2**. The growth of cells receiving vehicle is set as 100%, upon which the viability of cells treated with ERα17p and peptide **2** is calculated. Values shown are mean ± SD of three independent experiments performed in triplicate. (*) *p* < 0.05.

To confirm the involvement of GPER, the peptides ERα17p and **2** were tested at different concentrations, in wild type (WT) and GPER knockout (KO) MDA-MB-231 triple negative breast cancer cells. GPER knockout (KO) MDA-MB-231 cells were generated by CRISPR/Cas9-mediated genome editing (Fig. 6a,b). Remarkably, ERα17p and peptide **2** were able to reduce the viability of WT MDA-MB-231 but were inactive on GPER KO cells (Fig. 6c).

### The PLMI fragment induces similar anti-hyperalgesic effects than ERα17p and can be used at higher dose in vivo

**Figure 7:**
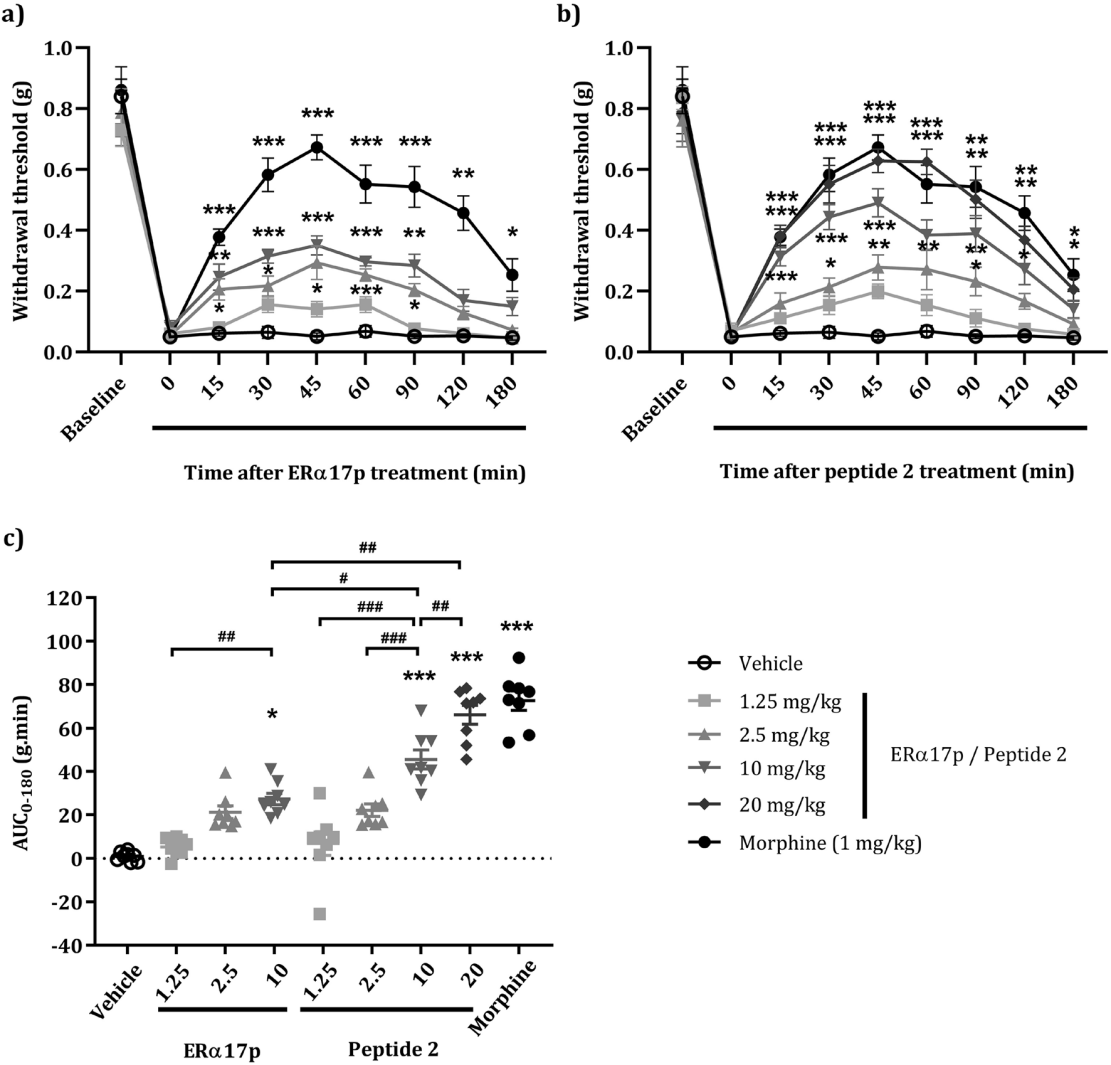
Effects of ERα17p and peptide 2 on tactile hypersensitivity in the murine CFA model by von Frey test. 50% paw withdrawal threshold (PWT) is determined using an adaptation of the Dixon up–down method. Seven days after CFA injection, anti-hyperalgesic actions of (**a**) ERα17p and (**b**) peptide **2** are evaluated by measuring dose-dependent effects. Von Frey test is assessed before CFA injection (baseline) and after vehicle injection (saline solution, control), ERα17p (1.25, 2.5 and 10 mg/kg), peptide **2** (1.25, 2.5, 10 and 20 mg/kg) or morphine (1 mg/kg, reference) treatments (i.p 10 mL/kg). The nine treatments of each experimental series (n = 8 mice *per* condition) are done in the same frame. Each treatment corresponds to a time-course curve. (**c**) Area under the time-course (0–180 min) of PWT variations from (**a**) and (**b**). Data are shown as mean ± SEM (n = 8 *per* group). *p < 0.05, **p < 0.01, ***p < 0.001, compared with the vehicle group; ^#^*p* < 0.05, ^##^*p* < 0.01, ^###^*p* < 0.001; two way ANOVA followed by Tukey test for the dose–response and Kruskal–Wallis followed by Dunn's test for AUC means comparison.

In vivo experiments were performed according to ethical guidelines established by the International Association for the Study of Pain (IASP) and the relevant European legislation (Directive 2010/63/EU). They were also approved by the Auvergne Animal Experiment Ethics Committee, CE2A and the French Ministry of Higher Education and Innovation. Compound **2**-mediated anti-hyperalgesic effects were compared to ERα17p by using von Frey test in Complete Freund's Adjuvant (CFA) model. Morphine (1 mg/kg) and vehicle (saline solution) were used as positive control and as reference, respectively. Mechanical paw withdrawal threshold (PWT) was used to evaluate pain. For each condition, eight mice were used. For all mice (Fig. 7a, n= 40), a decrease of the PWT from 0.79 ± 0.029 to 0.058 ± 0.0053 g (*p* < 0.001, *t*-test) was recorded, seven days after CFA injection. These data are relevant to the hyperalgesia symptoms encountered during chronic inflammation.

In the case of ERα17p, an increase of the PWT was observed at 45 min (0.051 ± 0.011 g for the vehicle *versus* 0.141 ± 0.025 g for 1.25 mg/kg of ERα17p and 0.293 ± 0.055 g for 2.5 mg/kg of ERα17p). A plateau was reached from 2.5 mg/kg of ERα17p with a maximum effect at 45 min (Fig. 7a). Due to its poor solubility, ERα17p was not tested at 20 mg/kg.

As for the peptide **2**, a dose-dependent increase of the PWT was also observed. At the dose of 20 mg/kg, PWT (0.628 ± 0.038 g) was not statistically different from the one obtained with 1 mg/kg of morphine (0.672 ± 0.041 g, *p* = 0.96), as shown in the Fig. 7b.

The anti-hyperlagesic action displayed by the two peptides was confirmed by the area under the time-course curve (AUC, in g.min, Fig. 7c). The calculated EC_50_ was 3.7 ± 1.9 mg/kg for ERα17p and 13.3 ± 6.7 mg/kg for the peptide **2**. Based on the molar concentrations of ERα17p (MW = 1900.36 g/mol) and of the peptide **2** (MW = 472.65 g/mol), the analgesic effects obtained with 10 mg/kg ERα17p (5.26 10^−6^ mol/kg) were similar to those effects obtained with **2** at 2.5 mg/kg (5.28 10^−6^ mol/kg).

## Discussion

According to docking studies, the N-terminal PLMI motif of the ERα17p synthetic peptide engulfs in the core of the GPER ligand-binding pocket, whereas the C-terminal counterpart (NSLSLALT motif) is packed at the entrance of the same site^23^. Thus, we have hypothesized that the PLMI motif could support the whole action of the full-length peptide, whereas the NSLALSLT motif could be responsible for the aggregation properties of ERα17p.

The propensity of ERα17p as well as of the peptides **1** (H_2_N-NSLALSLT-COOH) and **2** (H_2_N-PLMI-COOH) to generate aggregates was studied as a function of time, not only at different peptide concentrations but also at different pH. At pH 3.4, ERα17p remained soluble. At pH 7.4 and 9.1, a time-dependent exponential increase of the amount of aggregates with, however, a preference for pH 7.4 and elevated concentrations was observed. Accordingly, TEM images revealed different types of aggregates, depending on the pH. At pH 7.4, a dense network resembling to prefibrillar oligomers was shown, whereas mature amyloid fibrils were preferred in basic conditions. These observations were strengthened by NMR data recorded over a longer period (65 h *versus* 40 h). No plateau was reached at pH 7.4, even after 65 h, suggesting a slow and complex process with different types of aggregates. In this regard, we have shown in previous studies that ERα17p, which is random coil in solution, is able to fold into a β-strand regular structure to form a variety of regular spheres with a diameter ranging from 30 to 700 nm or to form a dense and rigid hydrogel, depending on experimental conditions^10,36,37,46,47^. This last remark could explain the absence of plateau at pH 7.4.

To decipher the part of ERα17p governing the formation of aggregates, we have studied the fibrilization properties of the fragments **1** and **2** flanking the soluble basic block KRSKK. The KRSKK motif was deleted from the primary sequence of the peptides of interest as it could increase their solubility and, therefore, introduce a bias. At pH 9.1 and after an incubation period of 18 h, the peptide **1** at 100 μM started to form fibrillar aggregates. TEM images failed to reveal twisted fibrils, in the contrary to ERα17p, making amyloid fibrils unlikely^36^. This observation corroborates the random coil CD signature. Indeed, signal intensity decreased rapidly to reach a negative maximum at ~ 24 h, confirming the formation of aggregates. The propensity of **1** to form aggregates could result from the motif SLALSLT (xLxLxLx sequence signature), which shares structural analogies with amyloidogenic ethylene responsive factors (ERF)-associated amphiphilic repression (EAR) motifs^48^. The peptide **2** being devoid of any aggregation properties, we assume that the C-terminal part of ERα17p (peptide **1**), only, is responsible for aggregation.

Then, we have explored the biological effects of the peptide **2**, not only on cell survival (in vitro study) but also in pain (in vivo study). Due to its poor solubility and its high propensity to precipitate in culture media, **1** was not tested. Our approach consisting in designing a minimalist active ERα17p-derived peptide, the pharmacologically inert KRSKK motif was excluded from our study^49^. Strikingly, peptide **2** interferes strongly with the survival of MCF-7 (ER+ , PR + , Her2 + , GPER +) and HS578T (ER-, PR-, Her2-, GPER+) cells, with an IC_50_ = 18 μM. Regarding phenotypes, a GPER-dependent mechanism is strongly likely. To confirm the involvement of GPER, we have compared the antiproliferative effects of ERα17p and **2** in GPER-positive (WT) and GPER knockout (KO) MDA-MB-231 cells obtained by the CRISPR/Cas9 gene editing technique. Remarkably, ERα17p and **2** were active in WT cells, only, confirming a GPER-dependent mechanism. It is of note that the pivotal role of GPER in the mechanism of action of ERα17p has been demonstrated elsewhere^23^. Response differences between MDA-MB-231, MCF-7 and HS578T cells could be explained by their respective phenotype. In the same context, the antiproliferative action of ERα17p at concentrations for which aggregation should be logically observed could result from differences between biophysical and biological experimental conditions. Lastly, it should be stressed that a role of the cellular uptake in ERα17p biological response seems unlikely, the GPER ligand-binding site being located close to the extracellular face of the protein^24,50,51^. As a matter of fact, ERα17p has been reported to be poorly internalized^46,49^.

In a last part of this work, we have compared the anti-hyperlagesic action of **2** to that of ERα17p. Considering the difference in molecular weight between the two peptides, ERα17p (MW = 1900.36 g/mol) activity at 10 mg/kg (i.e., 5.26 10^−6^ mol/kg) was similar to that of peptide **2** (MW = 472.65 g/mol) at 2.5 mg/kg (i.e., 5.28 10^−6^ mol/kg). These results are in accordance with cell growth data obtained with MDA-MB-231. The only difference between the two peptides is that the full-length analogue reaches a plateau at lower concentration than compound **2**, an observation relevant to the ability of the former to form pharmacologically inert aggregates^37^. However, we cannot exclude a contribution of bioavailability, metabolism and pharmacokinetics parameters. Hence, peptide **2** seems to support the whole intrinsic pharmacological activity of ERα17p.

By using ThT fluorescence spectroscopy, ^1^H-NMR and TEM, we have demonstrated that the formation of peptide ERα17p aggregates is not only concentration and time-dependent, but also pH-dependent. As such, ERα17p could form aggregates in neutral and basic but not in acidic cellular compartments. We have also evidenced that the aggregation properties of ERα17p are supported by the motif NSLALSLT in C-terminus, whereas the N-terminal PLMI motif is responsible for its pharmacological action. Since the PLMI tetrapeptide fails to generate aggregates, it could be much more advantageous to use it, instead of ERα17p, in a pharmaceutical context. Accordingly, the anti-nociceptive activity of ERα17p reaches a maximum at the dose of 2.5 mg/kg, whereas the PLMI fragment remains active to the dose of 20 mg/kg. The PLMI motif supporting the pharmacological action of the whole peptide, it should be considered not only as a hit for the design of new GPER modulators with dual antiproliferative and anti-nociceptive action, but also as a part of a putative ERα platform for the recruitment of GPER^52^.

## Methods

### Chemistry

Peptides (scale: 0.1 mmol, Boc strategy) were synthesized on an automated peptide synthesizer 433A (Applied Biosystems, Foster City, USA) by using a Boc-Thr(Bz) PAM resin (substitution range: 0.6–1.2 mmol.g^−1^). Dicyclohexylcarbodiimide (DCC) and hydroxybenzotriazole (HOBt) were used as coupling reagents. HF was used to cleaved the peptides from the resin. The C- and N-extremities were kept free. Purification was carried out by RP-HPLC by using a Waters setup comprising a Waters 1525 binary pump system and a Waters 2487 dual wavelength absorbance detector (Saint-Quentin en Yveline, France). UV detection was performed at 220 nm. Semi-preparative RP-HPLC was performed with an ACE 5 Å C8 column (10 × 250 mm) and a flow rate of 5 mL.min^−1^. Analytical RP-HPLC was performed with a Higgins Analytical RP proto 200 C18 5 µM column (4.6 × 100 mm) and a flow rate of 1 mL.min^−1^. Eluents were composed of appropriate percentages of solvent A (0.1% CF_3_COOH in H_2_O) and B (0.1% CF_3_COOH in CH_3_CN:H_2_O 60:40). MALDI-TOF mass spectrometry analysis was made on an ABI Voyager DE-Pro MALDI-TOF mass spectrometer (Applied Biosystems, Foster City, USA) by using as matrix a saturated solution of α-cyano-4-hydroxycinnamic acid (CHCA) in CH_3_CN:H_2_O:CF_3_COOH 50:50:0.1. Peptide **1** (H_2_N-NSLALSLT-COOH): Preparative RP-HPLC gradient: 5% to 60% (solvent B) over 10 min. R_t_ = 6.94 min. Calculated isotopic m/z = 817.44 (Found [M + H]^+^  = 818.47). Purification conditions and characterization of the peptides ERα17p (H_2_N-PLMIKRSKKNSLALSLT-COOH) and **2** (sequence: H_2_N-PLMI-COOH) were published elsewhere^46,49^.

### Fluorescence spectroscopy

Fibrils were detected by measuring the fluorescence intensity of Thioflavin T (ThT)^53^. A plate reader (Fluostar Optima, BMG LabTech, Ortenberg, Germany) and standard 96-wells flat-bottom black microtiter plates (Costar 3792, Sigma Aldrich, Saint-Louis, USA) were used in combination with excitation and emission filters, at 440 and 485 nm, respectively (ThT concentration: 10 μM). Peptide concentrations varied from 10 to 100 μM, at pH 3.4 (0.2 M glycine HCl in water), pH 7.4 (0.2 M KH_2_PO_4_ in water, 0.2 M K_2_HPO_4_, in water) and pH 9.1 (0.2 M glycine NaOH in water). The microtiter plate was shaken prior to measurements. Fluorescence intensity (arbitrary units, u.a.) was recorded every 10 min, during 40 h. Resulting curves were analyzed by using GraphPad Prism. Data are the means of experiments performed in triplicate.

### Nuclear magnetic resonance (NMR)

ERα17p fibrilization was followed at 298 K by using a NMR spectrometer (Bruker AVANCE III 500 MHz) equipped with a triple resonance (^1^H, ^15^ N, ^13^C) cryoprobe. Each sample (100 μM) was prepared extemporaneously in appropriate buffer at pH 3.4, 7.4 and 9.1. A 10:90 D_2_O:H_2_O mixture was added to the buffer to reach a final D_2_O percentage of 7.5%. The final volume was of 520 μL in a 5 mm tube. 1D proton experiments (eight scans) were carried out over 65 h, with a total of 258 spectra (1 spectrum each 15 min, 8 scan/spectrum). Spectra analysis was carried out with the Bruker Topspin software by focusing between 0.80 and 3.60 ppm aliphatic side-chains region.

### Transmission electron microscopy (TEM)

Peptides were dissolved in appropriate buffer at pH 3.4, 7.4 and 9.1 to reach peptide stock solutions of 100 μM. Each peptide was incubated in an Eppendorf tube during 48 h. One drop (20 μL) of each peptide solution was adsorbed onto glow-discharged carbon coated 200 mesh copper grids. After 2 min, the grids were dried and negatively stained during 45 s with a 2.5% uranyl acetate solution. Grids were blotted, dried, and examined by using a Zeiss 912 Omega (Zeiss, Marly-le-Roi, France) scanning electron microscope operating at 80 kV or a JEOL 2100 HC (Croissy, France) scanning electron microscope operating at 200 kV.

### Circular dichroism (CD)

CD spectra were recorded on a Jasco J-815 apparatus (Lisses, France) using quartz cells (0.1 cm path length, Hellma GmbH, Müllheim, Germany). Each peptide was dissolved (final concentration: 100 μM) in appropriate buffer solution, at pH 9.1. After an equilibration period of 5 min at 25 °C, CD spectra were recorded over the 190 to 260 nm wavelength range. Measurements were carried out at 1 nm intervals. Data points were acquired every 0.5 nm in continuous scanning mode (speed = 20 nm.min^−1^, bandwidth = 1 nm). CD spectra were recorded after 0, 5, 22 and 28 h (unit: mean residue ellipticity [*θ*]_MRE_, in deg.cm^2^.dmol^−1^). Acquisitions were carried out using the software Spectra Manager. Recorded data (mean of five scans) were treated with GraphPad Prism. Buffer background was subtracted to avoid buffer and cell contributions.

### Cell cultures

MCF-7 (ER + , PR + , Her2 + , GPER +) and HS578T (ER-, PR-, Her2-, GPER +) cells were cultured in RPMI-1640 and DMEM medium (Life Technologies, Courtaboeuf, France), respectively, supplemented with 10% (v/v) fetal bovine serum (FBS), 100 U/mL penicillin and 100 µg/mL streptomycin. MDA-MB-231 triple negative breast cancer cells were obtained from the ATCC (Manassas, VA, USA). Cells were maintained in DMEM/F12 (Dulbecco’s modified Eagle’s medium) with phenol red, supplemented with 5% fetal bovine serum (FBS) and 1% penicillin/streptomycin (Thermo Fisher Scientific, Monza, Italy). Cells were grown in a 37 °C incubator with 5% CO_2_.

### CRISPR/Cas9-mediated GPER knockout

Short guide RNA (sgRNA) sequence targeting human GPER was designed using the E-CRISP sgRNA Designer (http://www.e-crisp.org/E-CRISP/) and was cloned into the pSpCas9 (BB)-2A-Puro (PX459) plasmid, as previously described^54^. The GPER sgRNA sequence used to generate the GPER knockout is as followed: sgGPER: 5′-GGTGACAGGCTGGTCACCGC-3′. Next, the vector with sgRNA was transiently transfected into MDA-MB-231 cells using Lipofectamine LTX (Life Technologies, Milan Italy). Two days after transfection, cells were selected via growth in a medium containing 1 µg/mL puromycin dihydrochloride (Sigma-Aldrich, Milan, Italy). After antibiotic selection, the puromycin-resistant colonies were picked and cultured in regular medium. Then, immunoblots for GPER protein were performed to evaluate the efficiency of the GPER knockout.

### Cell growth studies

Viable cells were counted through 3-(4,5-dimethylthiazol-2-yl)-2,5-diphenyl tetrazolium bromide (MTT) colorimetric assay (Sigma, Saint-Quentin Fallavier, France)^55^. MCF-7 and HS578T cells (12,000 and 6,000 cells/well, respectively) were seeded in a 96-well plate and incubated overnight prior to 48 h treatment with various concentrations of peptide, or with vehicle (glucose 5%/glacial acetic acid 0.05%/DMSO 0.6%). The drug-containing medium was replaced with 100 μL of medium supplemented with 20% MTT. Cells were incubated for 4 h at 37 °C. Then, a 10% SDS/10 mM HCl solution (100 µL) was added to each well. Absorbance was measured at 570 and 620 nm by using an Infinite 200™ spectrophotometer (Tecan, Männedorf, Switzerland). Cells viability was evaluated by calculating absorbance difference (ΔOD = Abs_570nm_ − Abs_620nm_), which is proportional to the number of viable cells. Results correspond to the percentage of viability of treated cells, relative to vehicle treated control cells (N = 2 or 3).

MDA-MB-231 WT and GPER KO cells were seeded in 24-well plates in regular growth medium. After cells attached, they were incubated in a medium containing 2.5% charcoal-stripped fetal bovine serum (FBS) and treated for 72 h either in the presence or absence of the tested molecules. Treatments were renewed every day. Cells were counted on day 4 using an automated cell counter (Life Technologies, Milan, Italy), following the manufacturer’s recommendations.

### In vivo studies

#### Animals

Experiments were performed according to ethical guidelines established by the International Association for the Study of Pain (IASP) and the relevant European legislation (Directive 2010/63/EU). They were also approved by the Auvergne Animal Experiment Ethics Committee, CE2A and the French Ministry of Higher Education and Innovation. Eight-week-old male C57BL/6j mice were purchased from Janvier Laboratories (Le Genest-Saint-Isle, France), housed under standard laboratory conditions (12 h light/dark cycle, temperature of 21 to 22 °C, 55% humidity under specific pathogen free conditions) and acclimatized for a week before testing. Food and water were available ad libitum.

#### Experimental protocol

Design, analysis and reporting were carried out in accordance with the ARRIVE guidelines^56–58^. To ensure the methodological quality of the study, Rice et al*.* recommendations were followed^59^. Animals were randomly divided into eight mice *per* group. To assess different treatment effects over the same time interval and to avoid, thereby, unverifiable and time-variable environmental influence, treatments were administered following the method of equal blocks. All experiments were performed in a quiet room by the same blinded experimenter. ERα17p and PLMI peptides were dissolved in a saline solution prior to intraperitoneal administration (10 mL/kg).

#### Inflammatory pain model

A persistent inflammatory pain model was produced by injection under brief anesthesia (2.5% isoflurane inhalation) of Complete Freund's Adjuvant (CFA, 10 µL) on the left ankle joint of mice^60^. CFA consisted of *Mycobacterium butyricum* (Difco Laboratories, Detroit, USA) dissolved in paraffin oil and saline (0.9% NaCl). The solution was autoclaved 20 min at 120 °C. Behavior tests were performed before and seven days after CFA injection.

#### Von frey test

On behavior testing day, the mice were placed individually in Plexiglas compartments (8 cm (L) × 3.5 cm (W) × 8 cm (D)) and on an elevated wire mesh platform to allow access to the ventral surface of the hindpaws, and were allowed to acclimatize for one hour before testing. Von Frey filaments (0.02 to 1.4 g) were applied perpendicularly to the plantar surface of the paw. Paw withdrawal and licking were considered as positive responses. 50% paw withdrawal threshold (PWT) was determined using an adaptation of the Dixon up–down method, as described previously^61^.

#### Statistical analysis

All data were analyzed using the Prism 8 software (GraphPad™ Software Inc., San Diego, CA). Data were tested for normality (Shapiro–Wilk test) and equal variance (Fisher test). For kinetic data, multiple measurements were compared with repeated measures (two-way ANOVA). Post hoc comparisons were performed by the Tukey’s test. The area under the curve (AUC, 0–180 min) of 50% mechanical threshold (individual values) was calculated by the trapezoidal rule (reference: PWT baseline after CFA injection (threshold at time T_0_)). The AUC of individual values is the sum of each area between experimental times from 0 to 180 min (equation: (time T − time before time T) × [(threshold at time T − threshold at time T_0_) + (thresholds obtained at time T_0_ or at time before time T − threshold at time T_0_)/2]). AUC was expressed as mean ± SEM (g × min). A Kruskal–Wallis test followed by the Dunn's post hoc test was performed to have a mean comparison of the area under the time-course curves (AUC). Statistical differences significant at *p* < 0.05.

## Supplementary Information

Supplementary Information.

## Data Availability

The authors confirm that the data supporting the findings of this study are available within the article.
