# Supplementary figures and images for: Identification of a human estrogen receptor α tetrapeptidic fragment with dual antiproliferative and anti-nociceptive action
Source: Sci Rep. 2023 Jan 24;13:1326. doi: 10.1038/s41598-023-28062-9 (PMC9873809; doi:10.1038/s41598-023-28062-9)

**Supplementary Information**

**Figure 1.** Original WB images

**
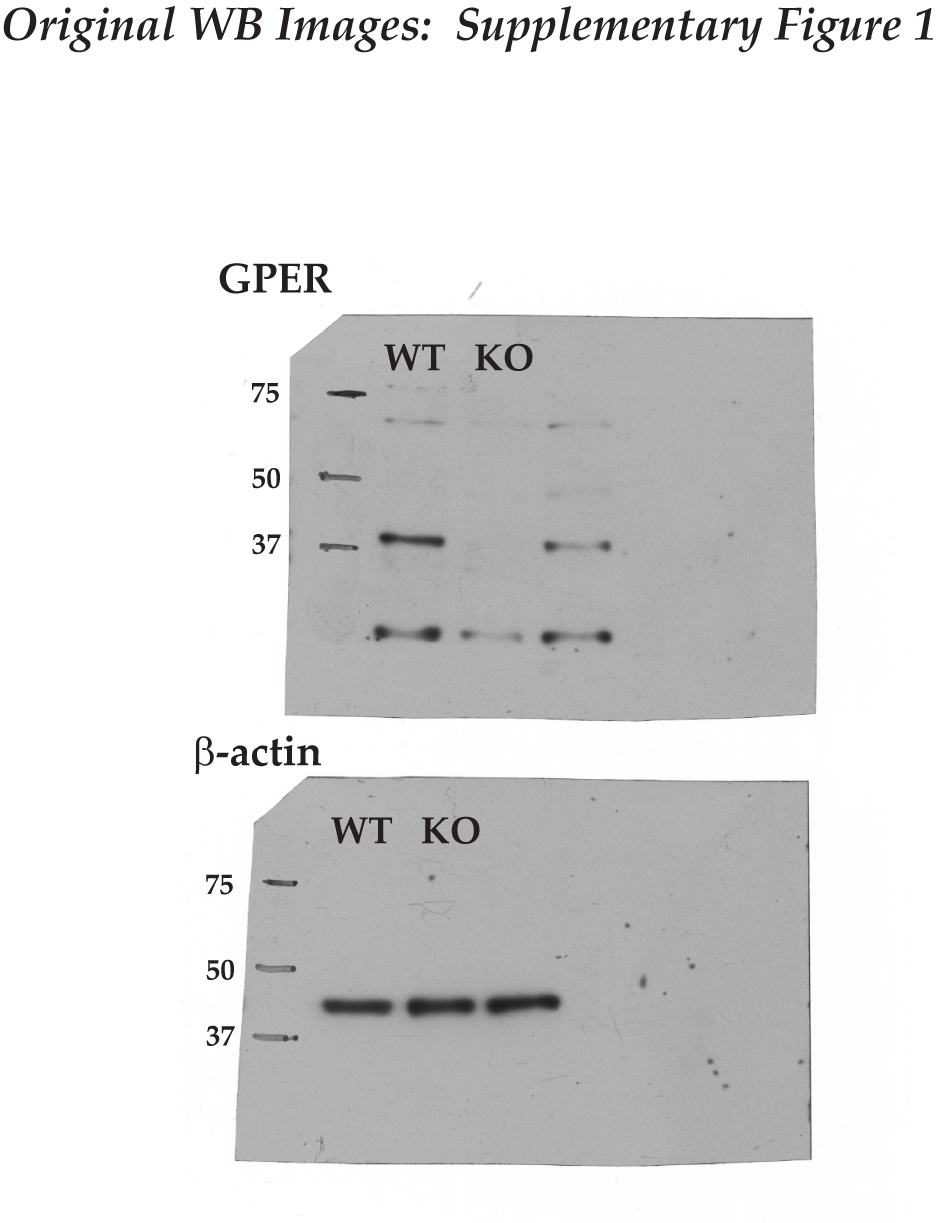
**

Supplement: Supplementary file 1 — Supplementary Information. [file 41598_2023_28062_MOESM1_ESM.docx]
